# Publisher Correction: Intron detention tightly regulates the stemness/differentiation switch in the adult neurogenic niche

**DOI:** 10.1038/s41467-024-47920-2

**Published:** 2024-04-24

**Authors:** Ainara González-Iglesias, Aida Arcas, Ana Domingo-Muelas, Estefania Mancini, Joan Galcerán, Juan Valcárcel, Isabel Fariñas, M. Angela Nieto

**Affiliations:** 1 Instituto de Neurociencias (CSIC-UMH), Sant Joan d’Alacant, 03550 Spain; 2 Departamento de Biología Celular, Biología Funcional y Antropología Física and Instituto de Biotecnología y Biomedicina, Universidad de Valencia, Burjassot, 46100 Spain; 3 Centro de Investigación Biomédica en Red sobre Enfermedades Neurodegenerativas (CIBERNED), 28029 Madrid, Spain; 4 Carlos Simon Foundation, 46980 Paterna, Valencia, Spain; 5 Centre for Genomic Regulation (CRG), The Barcelona Institute of Science and Technology, Barcelona, 08003 Spain; 6 Centro de Investigación Biomédica en Red sobre Enfermedades Raras (CIBERER), 28029 Madrid, Spain; 7 Universitat Pompeu Fabra (UPF), 08003 Barcelona, Spain; 8 Institució Catalana de Recerca i Estudis Avançats (ICREA), 08010 Barcelona, Spain; 9 Present Address: Department of Gene Therapy and Regulation of Gene Expression, Center for Applied Medical Research, University of Navarra, Pamplona, 31008 Spain; 10 Present Address: Department of Cell and Developmental Biology, Institute for Regenerative Medicine, Perelman School of Medicine, University of Pennsylvania, Philadelphia, PA 19104 USA; 11 Present Address: Igenomix Foundation, 46980 Paterna, Valencia, Spain

**Keywords:** RNA transport, Stem cells in the nervous system

Correction to: *Nature Communications* 10.1038/s41467-024-47092-z, published online 2 April 2024

In this article the affiliation details for Juan Valcárcel were incorrectly given as ‘Carlos Simon Foundation, 46980 Paterna, Valencia, Spain’, ‘Universitat Pompeu Fabra (UPF), 08003 Barcelona, Spain’ and ‘Institució Catalana de Recerca i Estudis Avançats (ICREA), 08010 Barcelona, Spain’ but should have been ‘Centre for Genomic Regulation (CRG), The Barcelona Institute of Science and Technology, Barcelona 08003, Spain’, ‘Universitat Pompeu Fabra (UPF), 08003 Barcelona, Spain’ and ‘Institució Catalana de Recerca i Estudis Avançats (ICREA), 08010 Barcelona, Spain’.

