# Supplementary material for: Intron detention tightly regulates the stemness/differentiation switch in the adult neurogenic niche
Source: Nat Commun. 2024 Apr 2;15:2837. doi: 10.1038/s41467-024-47092-z (PMC10987655; doi:10.1038/s41467-024-47092-z)
Supplement: Supplementary file 1 — Supplementary Information [file 41467_2024_47092_MOESM1_ESM.pdf]

Supplementary Information

Intron detention tightly regulates the stemness/differentiation switch in the adult neurogenic niche

Ainara González-Iglesias[1], Aida Arcas[1,6], Ana Domingo-Muelas[2,3,7], Estefania Mancini[4], Joan Galcerán[1,5], Juan Valcárcel[4], Isabel Fariñas[2,3], M. Angela Nieto[1,5,8*]

Correspondence to: anieto@umh.es

This PDF file includes:

Supplementary Figures 1-12

Supplementary Table 1

Supplementary figure 1

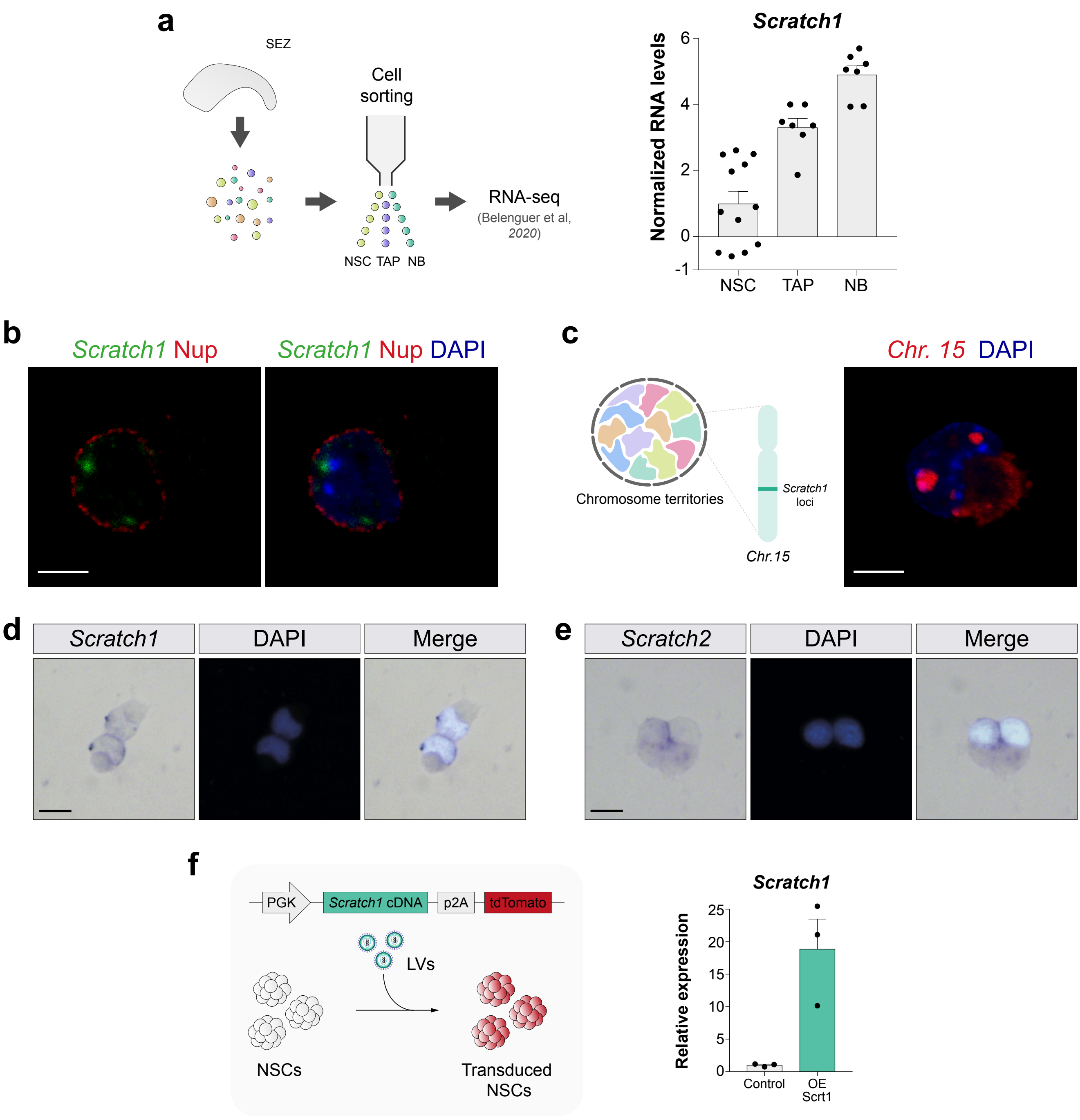

Supplementary figure 1| *Scratch1* mRNA accumulates in the nucleus of NSCs. a, Schematic representation of the isolation and RNA sequencing of different cell populations from the adult SEZ and relative *Scratch1* expression in NSCs, TAPs and neuroblasts (n=4 mice). **b**, Optical section of an *in situ* hybridisation for *Scratch1* mRNA (green) combined with immuno-histochemistry for nucleoporins (red) in cultured NSCs. **c**, DNA *in situ* hybridisation for chromosome 15 (red), where *Scratch1* locus is located, in cultured NSCs. **d**, Bright field image of *Scratch1* mRNA *in situ* hybridisation in NSCs in culture. **e**, Bright field image of *Scratch2* mRNA *in situ* hybridisation in NSCs in culture. **f**, Analysis of *Scratch1* overexpression efficiency in NSC cultures by qPCR (n=3 biologically independent samples). Scale bars: **b-c**, 5 µm; and **d-e**, 10 µm. NSC, neural stem cell; TAP, transient amplifying progenitors; NB, neuroblasts. Data are presented as mean values ± SEM. Source data are provided as a Source Data file.

Supplementary figure 2

a

Human

SCRATCH1

GGCGGGGGCGGAGCGTGGCCCGGCCCTGTCTCATCCTCTCCTCCCCTCTCTTCCCCTCCCTGCTGTCTCCATCCTCCCTG
CCCGGCGGTCCCTCGGCCTCCTCTCCTTTGTCCTCCTCCCTGTTCGCCTCTCTCCCGGACCCGGGTCTCCCGGTCCGCGT
CTGCTGTCTCCCGCCCCCCCATCCCCCGTGTGTGTCCTGCCGCCGCCCCATTCCCTTCCCGCTGTCCGTCCGCCTCCGCC
CGCAG

SCRATCH2

CGCAAAGGGTGACGCGCTCAAAGCGGGAAACCCTCTTCGGCGCCTCCTCTCACCGCCCCTCCACTTCTCGCCCCCTCCAG

Mouse

Scratch1

CTCTGCCCTGTGGTCCCCTGCTCCTTCTCCTTTGTCCTAATCCCTGTTTGCCTTCCTTTCGGACCCCGACGTCTCCCGCTGT
GTCTCCTCGATCTCCCGCCGCCCGCTTTGCGTGTGTCTTGCCCGCTGCTCCGTTCCCTTCCCGCTGTCTGTCTGTCCGCCT
CCGCCCTCAG

Scratch2

CGCACTGGTGTAGGGCGGCCTGCGGGGAACAACCCGCACCCCACCCCGGCCTCATGCCCCTCTTTGTCACCCGCAG

Zebrafish

scrt1a

CACTAATTGAAATGGAGAAAGCTGTTGATTTCTTGCAGAAATTAATAAATAACTGTGGCTTTGTGTTTTTTTTTTCCCAAG

scrt1b

ATCGCCGAACCCCATTGTCCTCAGCCGATTCCCTTGTGAATATGAATAATTGAATAAAAGATCTTGTTCTTTCCATGCTAG

scrt2

TTTACACTTGCAGATCAGTGCAGAAGTGAAAGGTCAATGAGTTAATAACACATTCATGTTCTCTGCCCGGCTGCAG

Branch point | Pyrimidines | 3' splice site

b

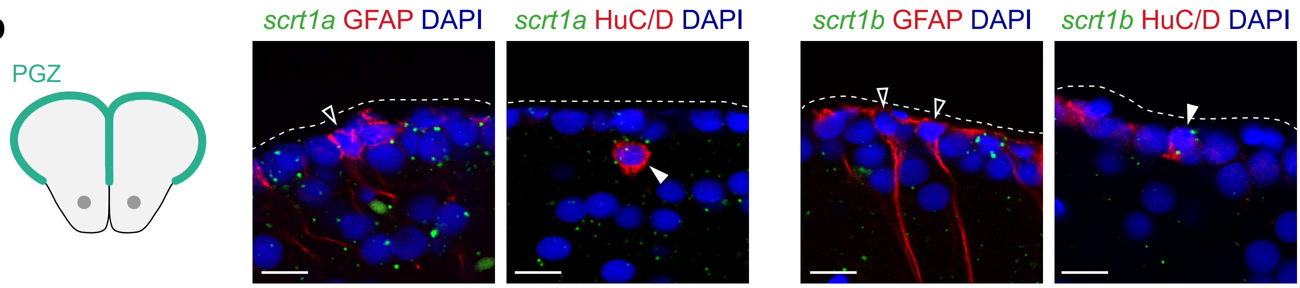

Supplementary figure 2| The regulation of *Scratch1* mRNA subcellular localization by intron retention is specific of the mammalian SEZ. a, Sequence comparison of polypyrimidine tracts in *Scratch1* and *Scratch2* introns in different species. **b**, Expression pattern of *scrt1a* (left panel) and *scrt1b* (right panel) in the PGZ of zebrafish: *In situ* hybridisation for *scrt1a* (left panel, green) combined with immunohistochemistry for GFAP (left, red) or HuC/D (right, red); and *in situ* hybridisation for *scrt1b* (right panel, green) combined with immunohistochemistry for GFAP (left, red) or HuC/D (right, red). Arrowheads point to double-positive cells, and empty arrowheads point to single-positive cells. Scale bars represent 10 μm.

Supplementary figure 3

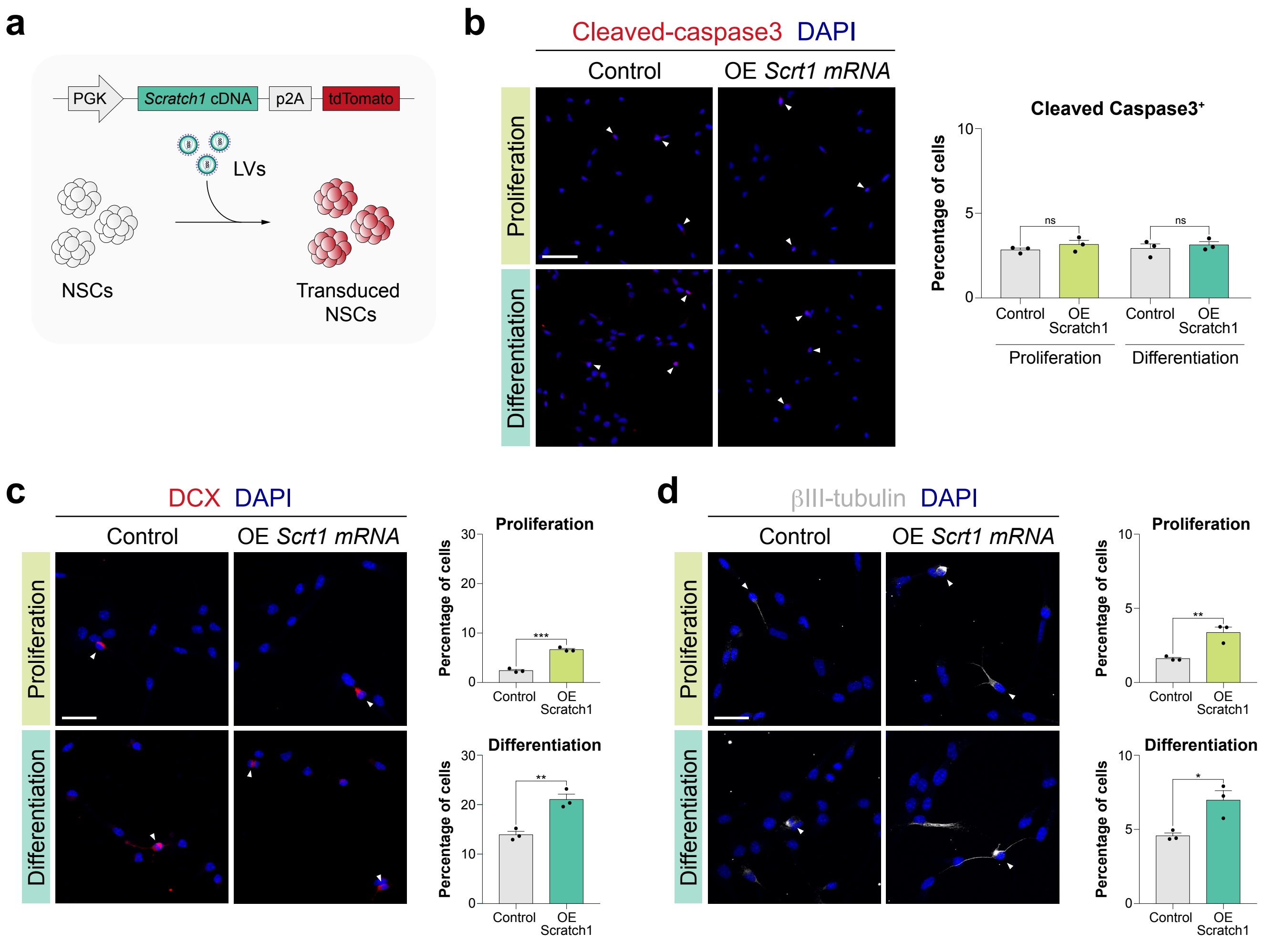

Supplementary figure 3| Scratch1 promotes the survival of the differentiating cells and their terminal differentiation into neurons. a, Schematic drawing representing the transduction of NSCs with lentiviruses (LV) and the construct use for *Scratch1* overexpression experiments. **b**, Immunodetection and quantification of cleaved-caspase3^{+} cells (red) in cultures of adult NSCs two days after plating the cells, both in proliferation and differentiation conditions (2DIV; *p*-value(proliferation)=0.293, *p*-value(differentiation)=0.572, n=3 biologically independent samples, by two-tailed Student's t-test). **c**, Immunodetection and quantification of DCX+ cells (red) in control or *Scratch1* overexpressing NSC cultures two days after plating the cells, both in proliferation and differentiation conditions (2DIV; *p*-value(proliferation)=0.0002, *p*-value(differentiation)=0.0051, n=3 biologically independent samples, by two-tailed Student's t-test). **d**, Immunodetection and quantification of βIII-tubulin+ cells (white) in control or *Scratch1* overexpressing NSC cultures two days after plating the cells, both in proliferation and differentiation conditions (2DIV; *p*-value(proliferation)=0.009, *p*-value(differentiation)=0.023, n=3 biologically independent samples, by two-tailed Student's t-test). Arrowheads point to positive cells. Scale bars represent 25 µm. Data are presented as mean values ± SEM. ns, not significant; *p < 0.05, **p < 0.01, ***p < 0.001. Source data are provided as a Source Data file.

Supplementary figure 4

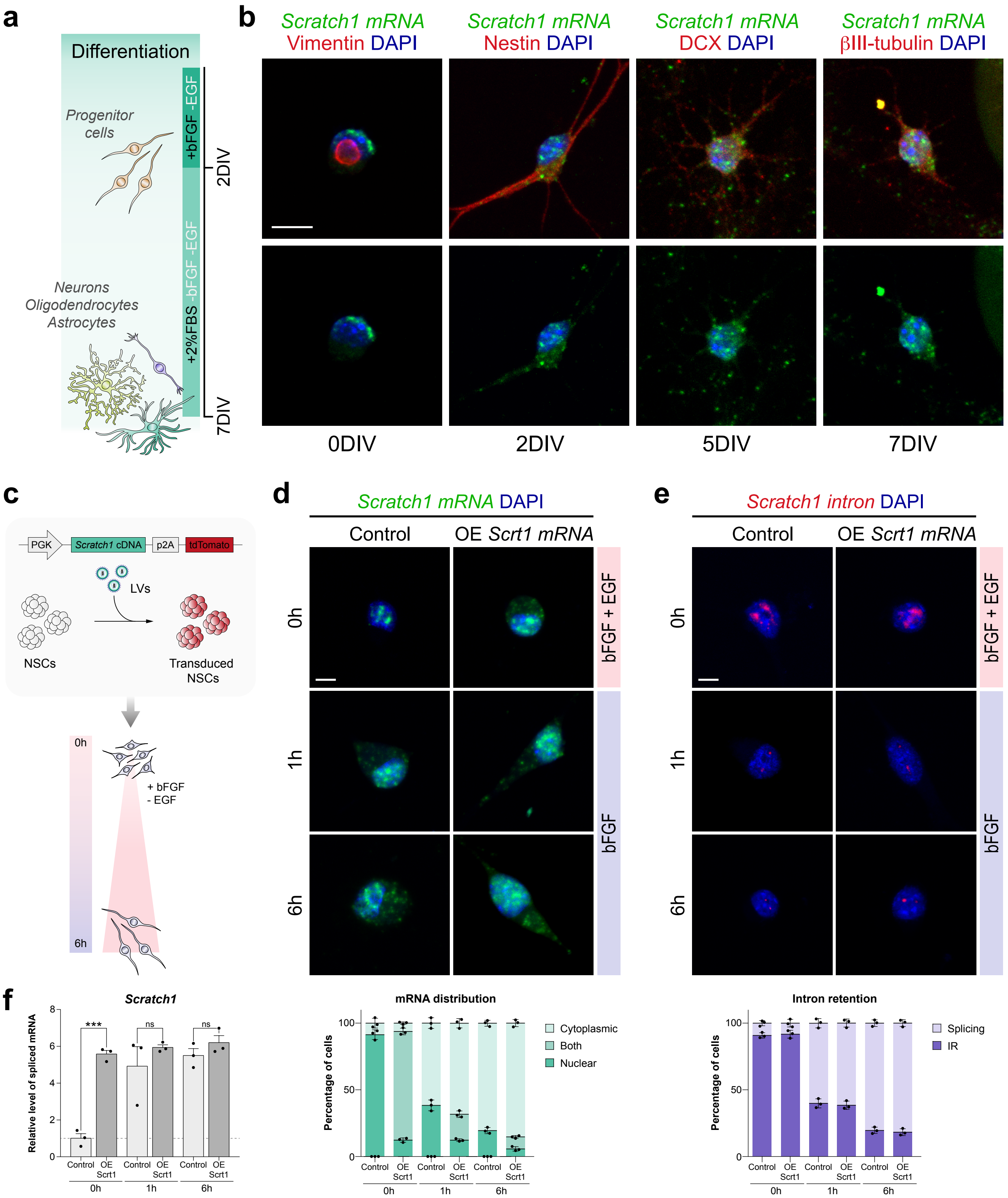

Supplementary figure 4| *Scratch1* mRNA presents a cytoplasmic distribution upon NSC differentiation. a, Schematic representation of the protocol used to induce the differentiation of NSCs in culture. **b**, *In situ* hybridisation for *Scratch1* (green), combined with immunohistochemistry for Vimentin, Nestin, DCX or βIII-tubulin (red), in NSC cultures at 0, 2, 5 and 7 days after the induction of differentiation. **c**, Schematic drawing representing the transduction of NSCs with lentiviruses (LV) and the construct use for *Scratch1* overexpression experiments. Transduced NSCs were subsequently cultured in differentiation conditions. **d**, *In situ* hybridisation for *Scratch1* mRNA (green) in control and *Scratch1* overexpressing NSCs at 0h, 1h and 6h after the induction of differentiation. Quantification of mRNA distribution (n=3 biologically independent samples). **e**, *In situ* hybridisation for *Scratch1* intron (red) in control and *Scratch1* overexpressing NSCs at 0h, 1h and 6h after the induction of differentiation. Quantification of intron retention (n=3 biologically independent samples). **f**, Ratio of spliced *Scratch1* mRNA in control and *Scratch1* overexpressing NSCs at 0h, 1h and 6h after the induction of differentiation (*p*-value(0h)=0.00014, *p*-value(1h)=0.39683, *p*-value(6h)=0.26206, n=3 biologically independent samples, by two-tailed Student's t-test). Scale bars represent 10 µm. Data are presented as mean values ± SEM. ns, not significant; $^{*}p < 0.05$, $^{**}p < 0.01$, $^{***}p < 0.001$. Source data are provided as a Source Data file.

Supplementary figure 5

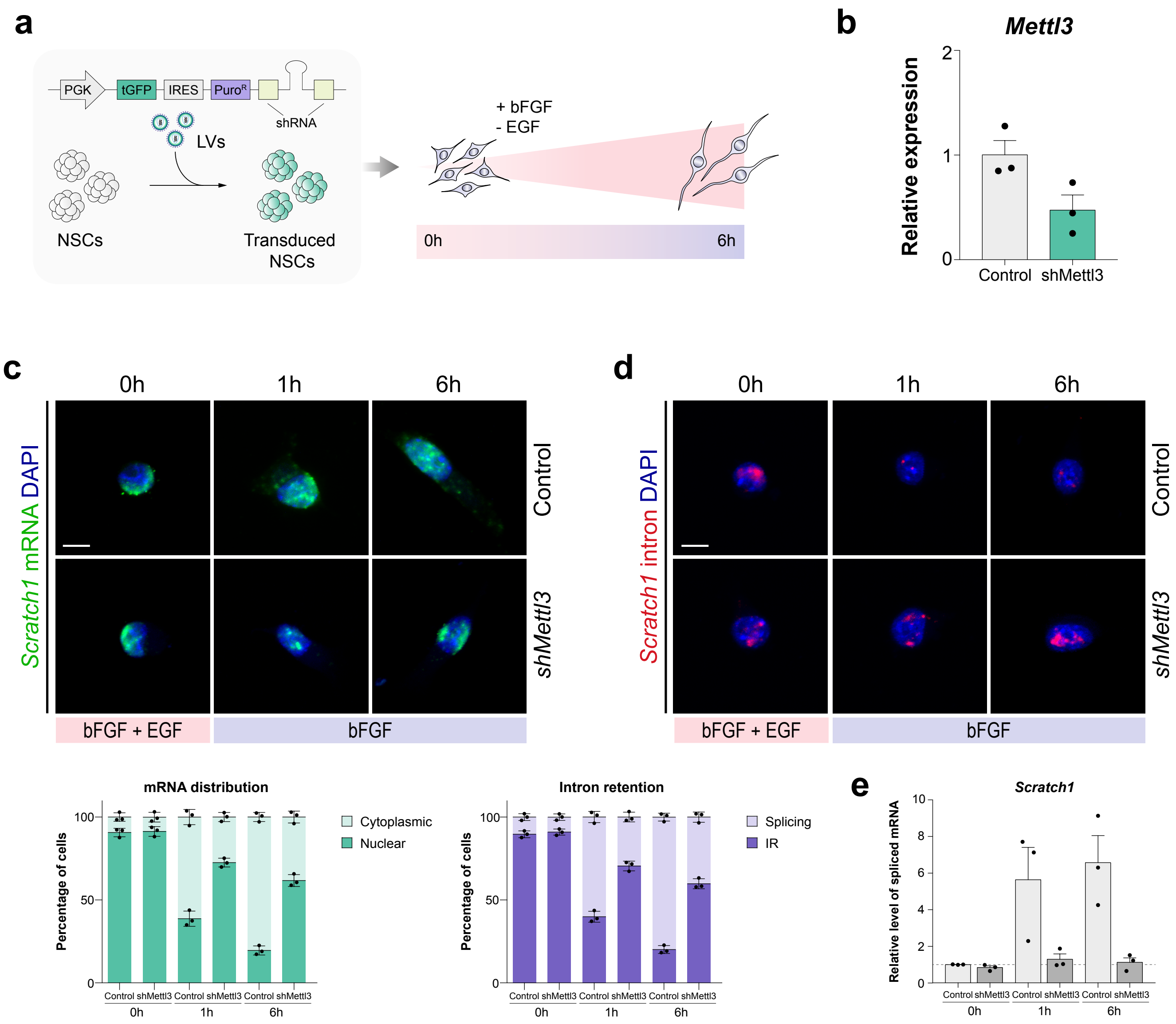

Supplementary figure 5| *Mettl3* loss of function prevents *Scratch1* mRNA splicing and export during NSC differentiation. a, Schematic representation of the transduction of NSCs with lentiviruses (LV) and the construct used for *Mettl3* loss of function experiments. Transduced NSCs were subsequently cultured either in differentiation conditions. **b**, Analysis of *Mettl3* shRNA efficiency in NSC cultures by qPCR (n=3 biologically independent samples). **c**, *In situ* hybridisation for *Scratch1* mRNA (green) in NSC cultures previously infected with control or shMettl3 lentiviruses at 0h, 1h and 6h after the induction of differentiation. Quantification of mRNA distribution (n=3 biologically independent samples). **d**, *In situ* hybridisation for *Scratch1* intron (red) in NSC cultures previously infected with control or shMettl3 lentiviruses at 0h, 1h and 6h after the induction of differentiation. Quantification of intron retention (n=3 biologically independent samples). **e**, Ratio of spliced *Scratch1* mRNA in control or shMettl3 NSCs at 0h, 1h and 6h after the induction of differentiation (n=3 biologically independent samples). Scale bars represent 5 μm. Data are presented as mean values ± SEM. Source data are provided as a Source Data file.

Supplementary figure 6

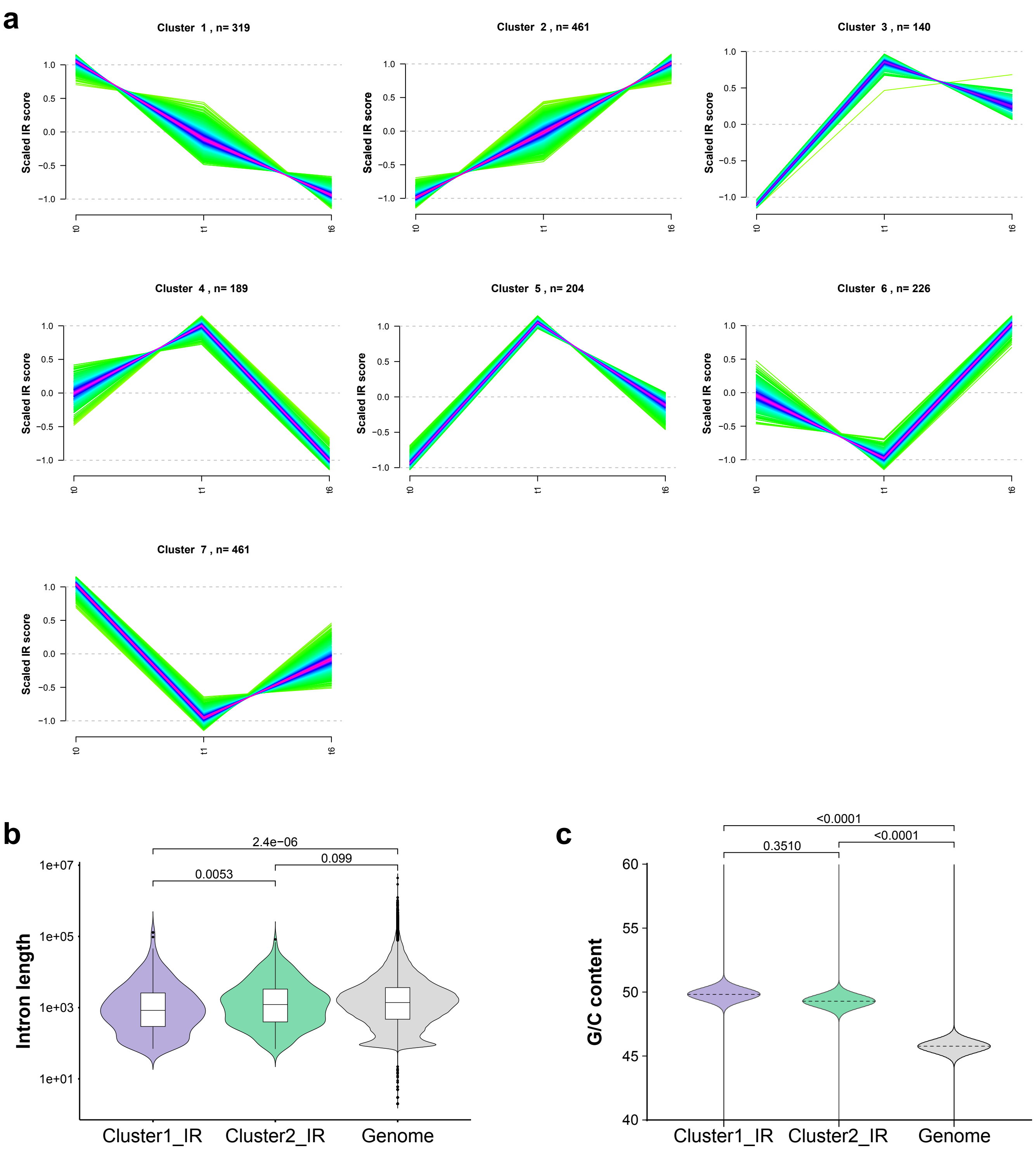

	Cluster1_IR	Cluster 2_IR	Genome
Mean	3,750	3,529	5,554

	Cluster1_IR	Cluster 2_IR	Genome
%GC	49.83%	49.30%	45.77%

Supplementary figure 6| Trends in intron detention during NSC differentiation. a, Plots depicting the different splicing patterns identified for all the intron detention events detected, grouped using soft clustering based on fuzzy c-means. **b-c**, Plots representing the comparison between Cluster1 retained introns, Cluster2 retained introns and all the introns in the genome regarding their length (**b**) and their G/C content (**c**). Source data are provided as a Source Data file.

Supplementary figure 7

Data from Baser *et al.*, (2019)*;* GSE944991

a

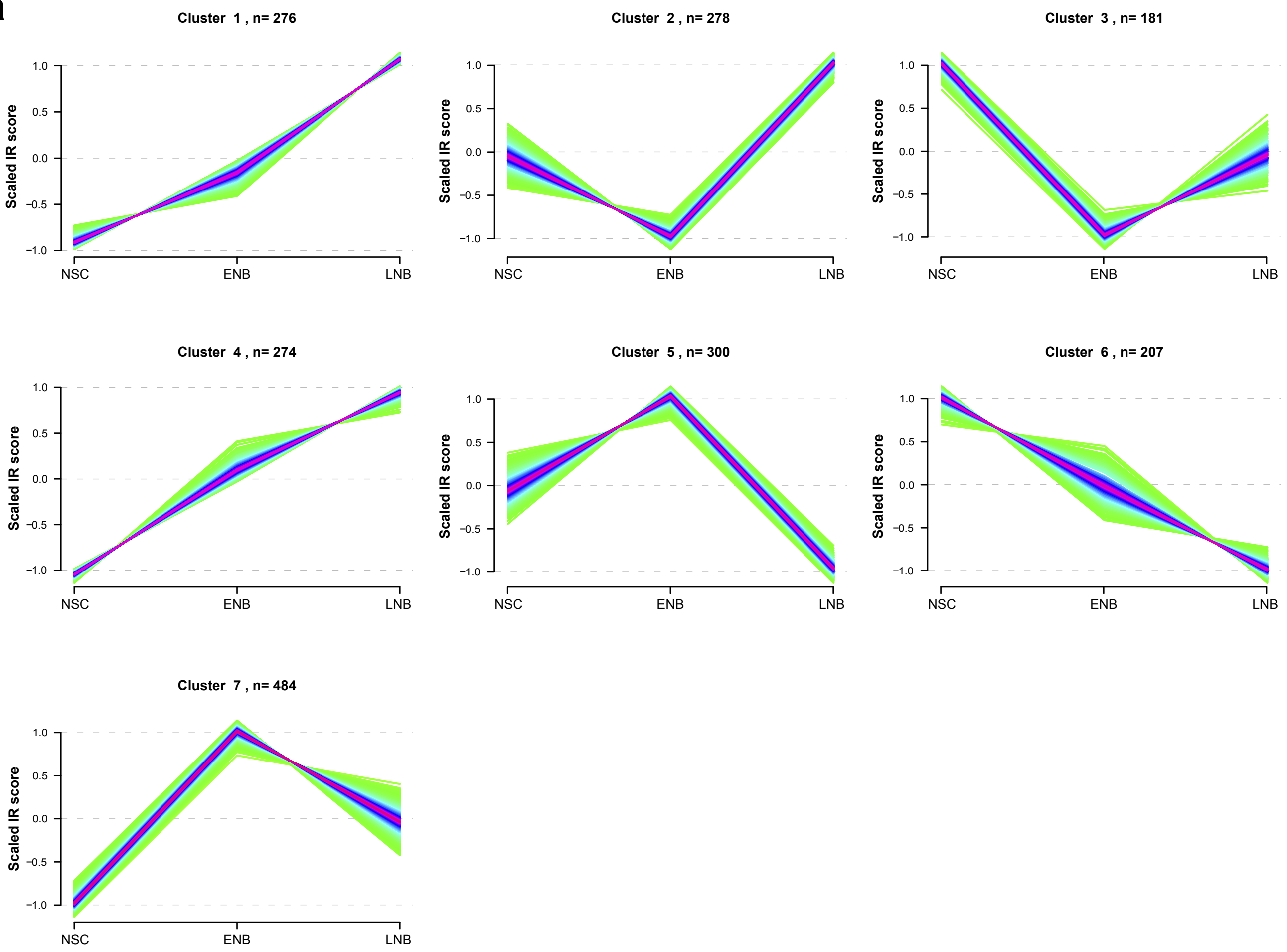

Cluster 1 + 4

b

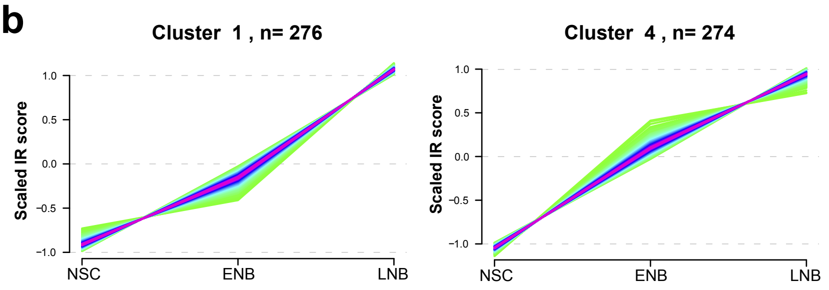

Regulation of stem cell population maintenance *Cnot1, Smg1, Ylpm1, Symd5, Xrn1, Map3k4*

MAPK cascade *Dvl3, Taok3, Map3k4, Lrrc7, Sos1, Sptan1*

Regulation of cell division *Setd2, Smyd5, Birc6, Rab11fip3, Cul3, Cit*

Negative regulation of neuron differentiation *Disp3, Nepro, Pcm1*

Cluster 6

c

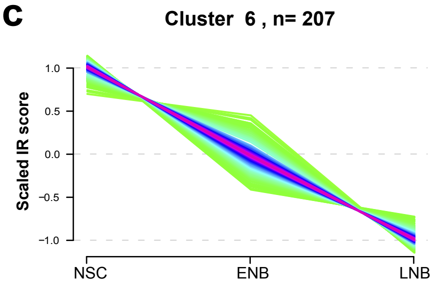

Neurogenesis *Plxna2, Ss18l1, Usp9x, Dchs1, Apc*

Neuron differentiation *Celsr2, Eif2ak4*

Nervous system development *Mast1, Apc, Pkd1, Dlg5, Ep300*

Axonogenesis *Sema5a, Macf1, Mycbp2*

Axon guidance *Robo3, Sema6c, Ablim2, Sema4d*

Supplementary figure 7| Trends in intron detention during NSC differentiation in the adult SEZ. RNA-seq data obtained from Baser et al. (2019). **a**, Plots depicting the different splicing patterns identified for all the intron detention events detected, grouped using soft clustering based on fuzzy c-means. **b**, Plots illustrating time point-specific changes in intron detention for genes belonging to Clusters 1 and 4 (left) and representative Gene Ontology (GO) terms of the biological process categories enriched in these clusters (right). **c**, Plot illustrating time point-specific changes in intron detention for genes belonging to Cluster 6 (left) and representative Gene Ontology (GO) terms of the biological process categories enriched in this cluster (right). Source data are provided as a Source Data file.

Supplementary figure 8

Cluster 1

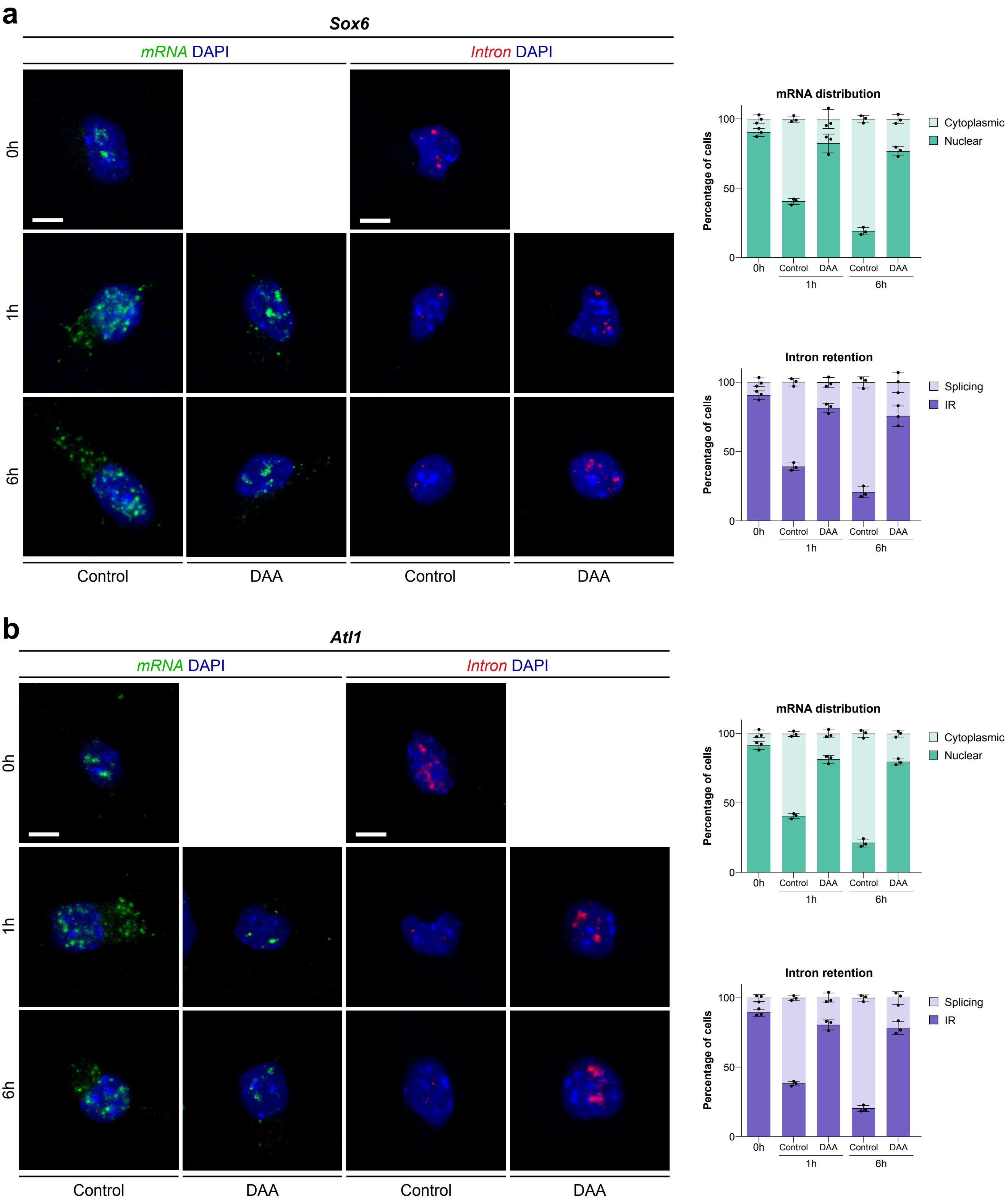

Supplementary figure 8| Validation of genes in Cluster 1. a, *In situ* hybridisation for *Sox6* mRNA (green) or intron (red) in control NSCs and in NSCs treated with DAA. Quantification of mRNA distribution and intron retention (n=3 biologically independent samples). **b**, *In situ* hybridisation for *Atl1* mRNA (green) or intron (red) in control NSCs and in NSCs treated with DAA. Quantification of mRNA distribution and intron retention (n=3 biologically independent samples). Scale bars represent 5 µm. Data are presented as mean values ± SEM. Source data are provided as a Source Data file.

Supplementary figure 9

Cluster 2

a

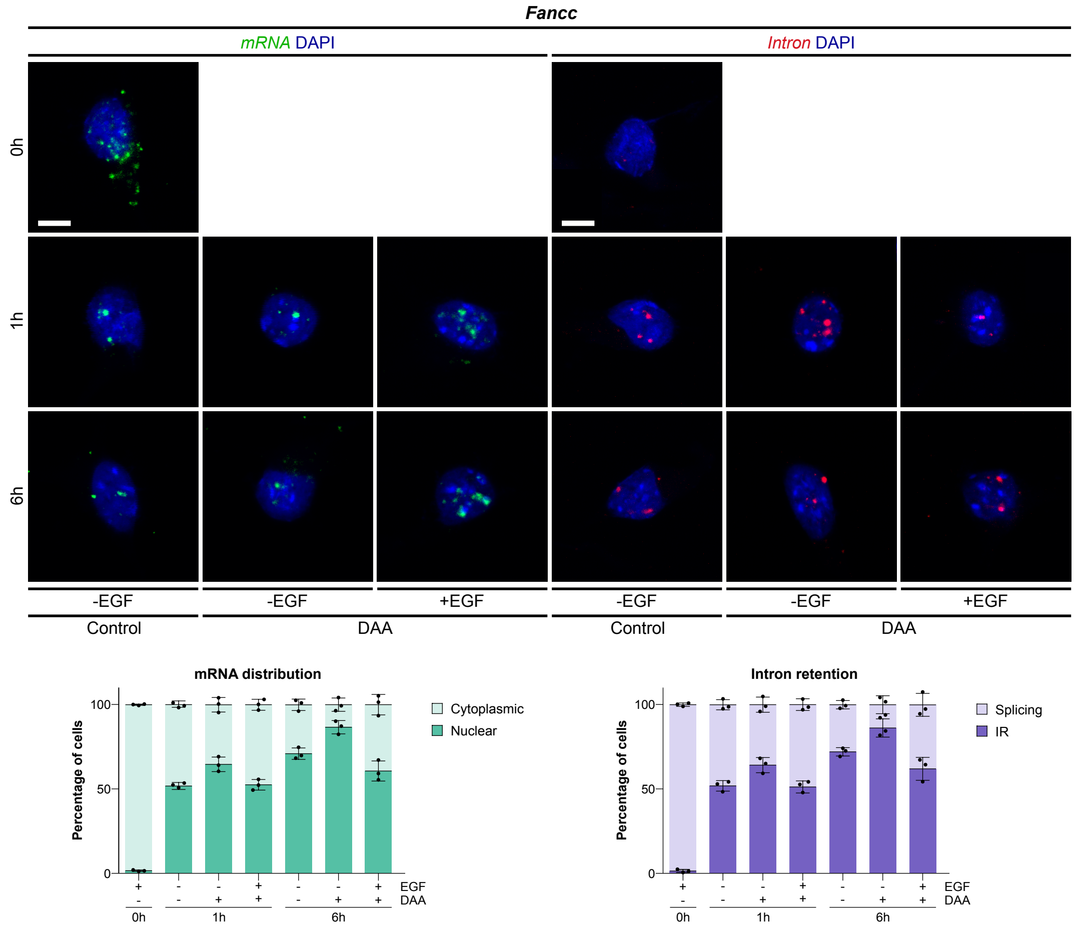

b

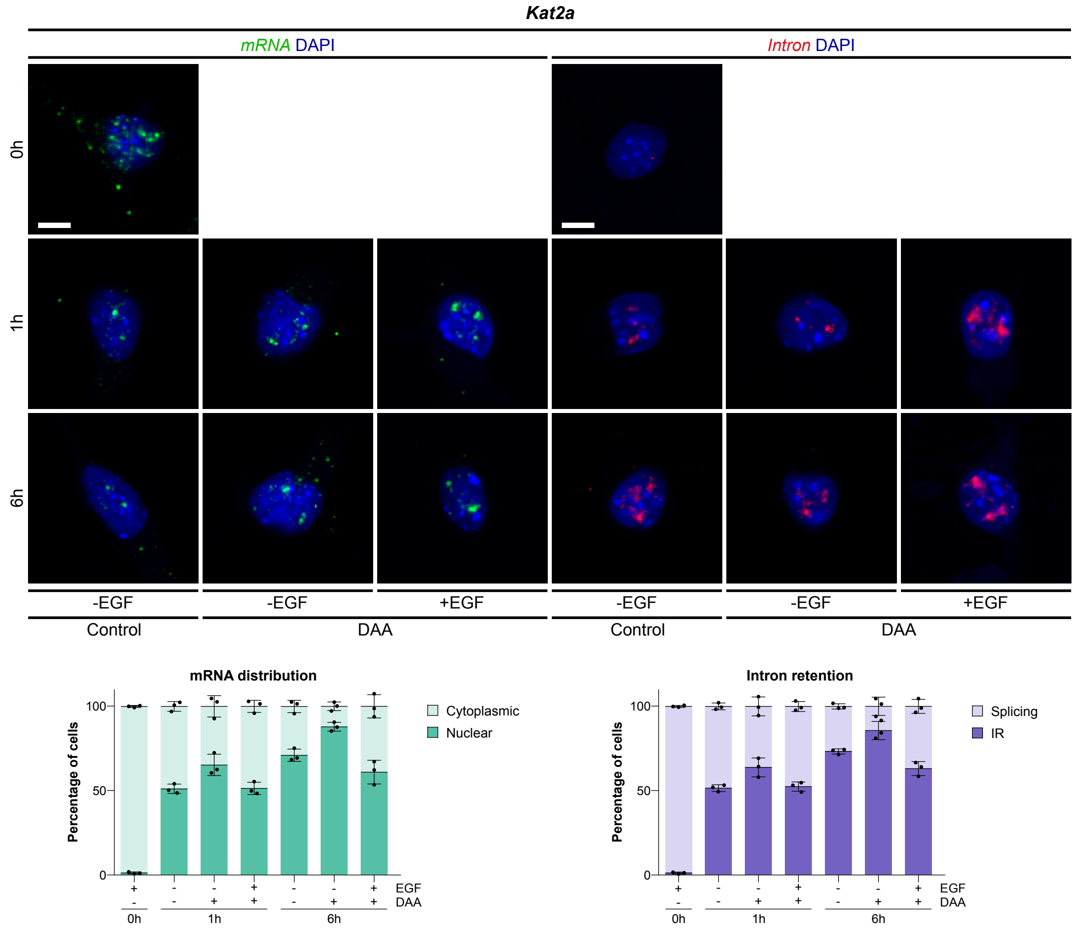

Supplementary figure 9| Validation of genes in Cluster 2. a, *In situ* hybridisation for *Fancc* mRNA (green) or intron (red) in control or DAA-treated NSCs. Quantification of mRNA distribution and intron retention (n=3 biologically independent samples). **b**, *In situ* hybridisation for *Kat2a* mRNA (green) or intron (red) in control or DAA-treated NSCs. Quantification of mRNA distribution and intron retention (n=3 biologically independent samples). Scale bars represent 5 μm. Data are presented as mean values ± SEM. Source data are provided as a Source Data file.

Supplementary figure 10

Cluster 1

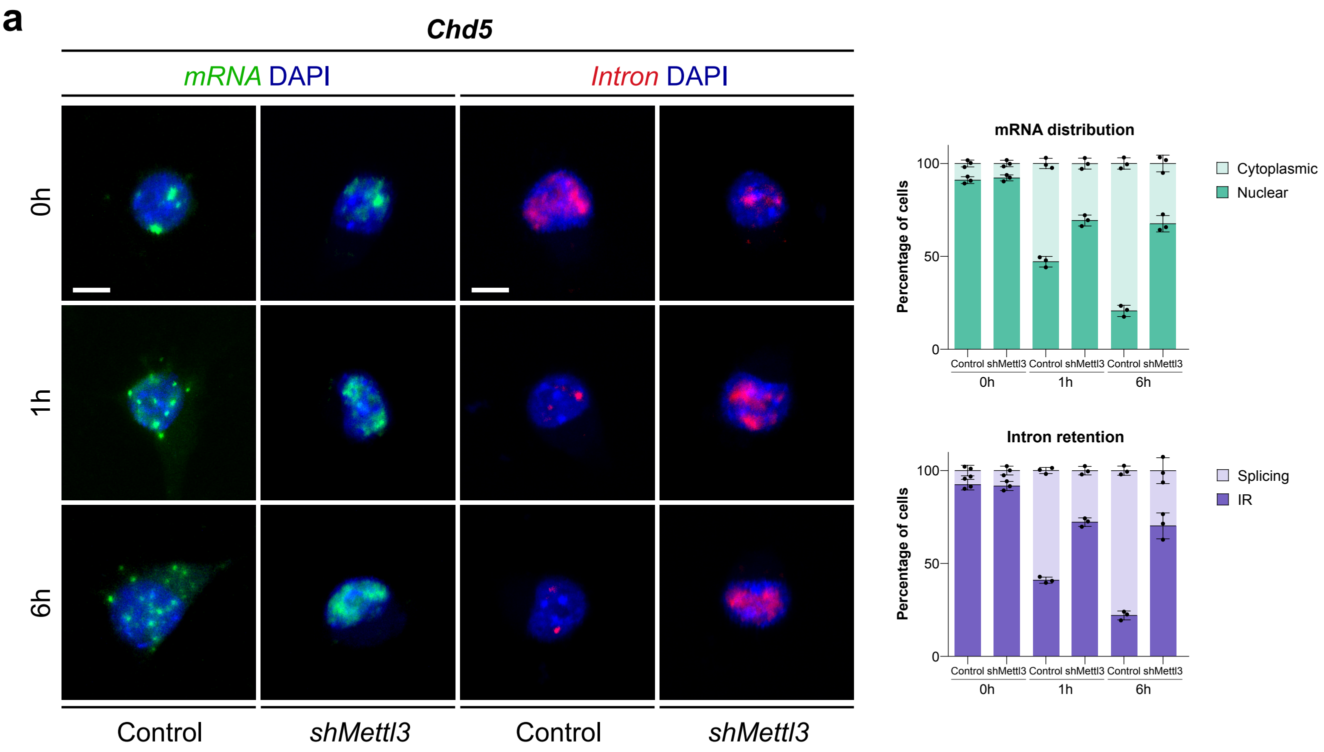

Cluster 2

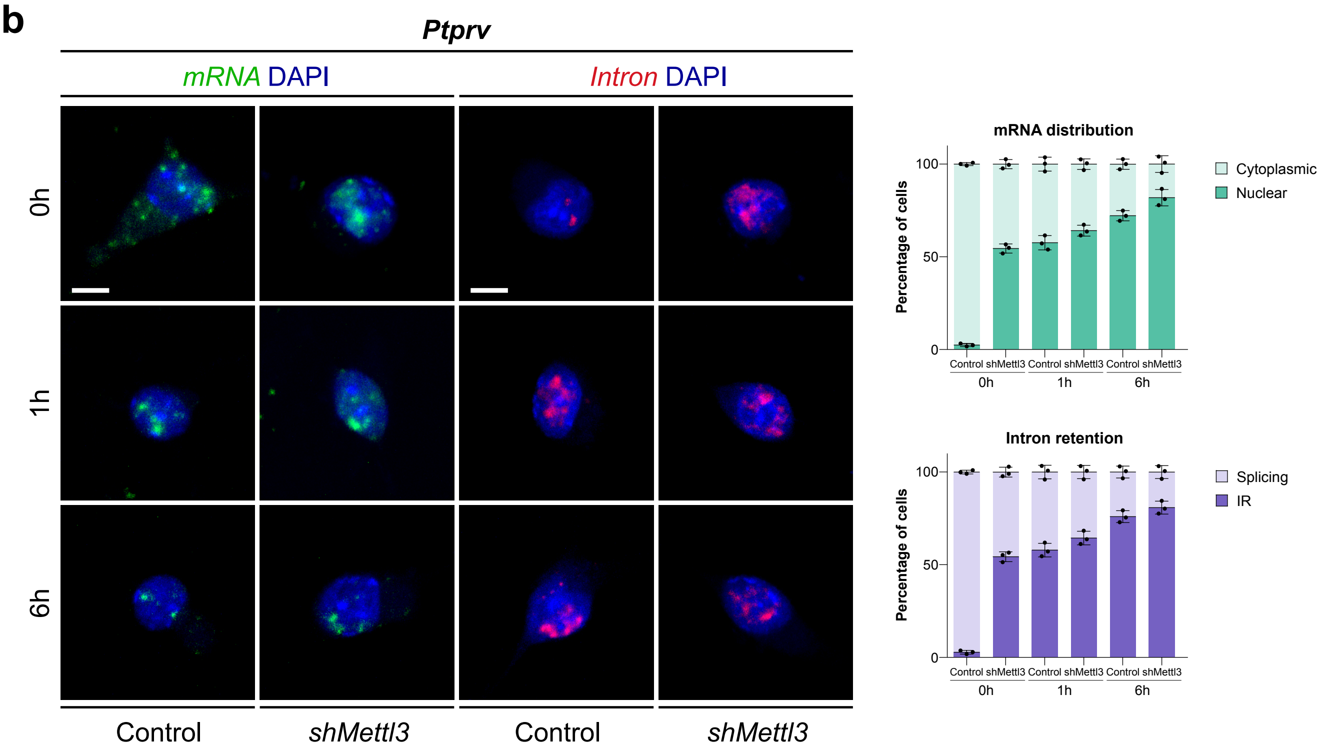

Supplementary figure 10| mRNA distribution and splicing status of genes in Cluster 1 and 2 after *Mettl3* loss of function. **a**, *In situ* hybridisation for *Chd5* mRNA (green) or intron (red) in NSC cultures previously infected with control or shMettl3 lentiviruses at 0h, 1h and 6h after the induction of differentiation. Quantification of mRNA distribution and intron retention (n=3 biologically independent samples). **b**, *In situ* hybridisation for *Ptprv* mRNA (green) or intron (red) in NSC cultures previously infected with control or shMettl3 lentiviruses at 0h, 1h and 6h after the induction of differentiation. Quantification of mRNA distribution and intron retention (n=3 biologically independent samples). Scale bars represent 5 µm. Data are presented as mean values ± SEM. Source data are provided as a Source Data file.

Supplementary figure 11

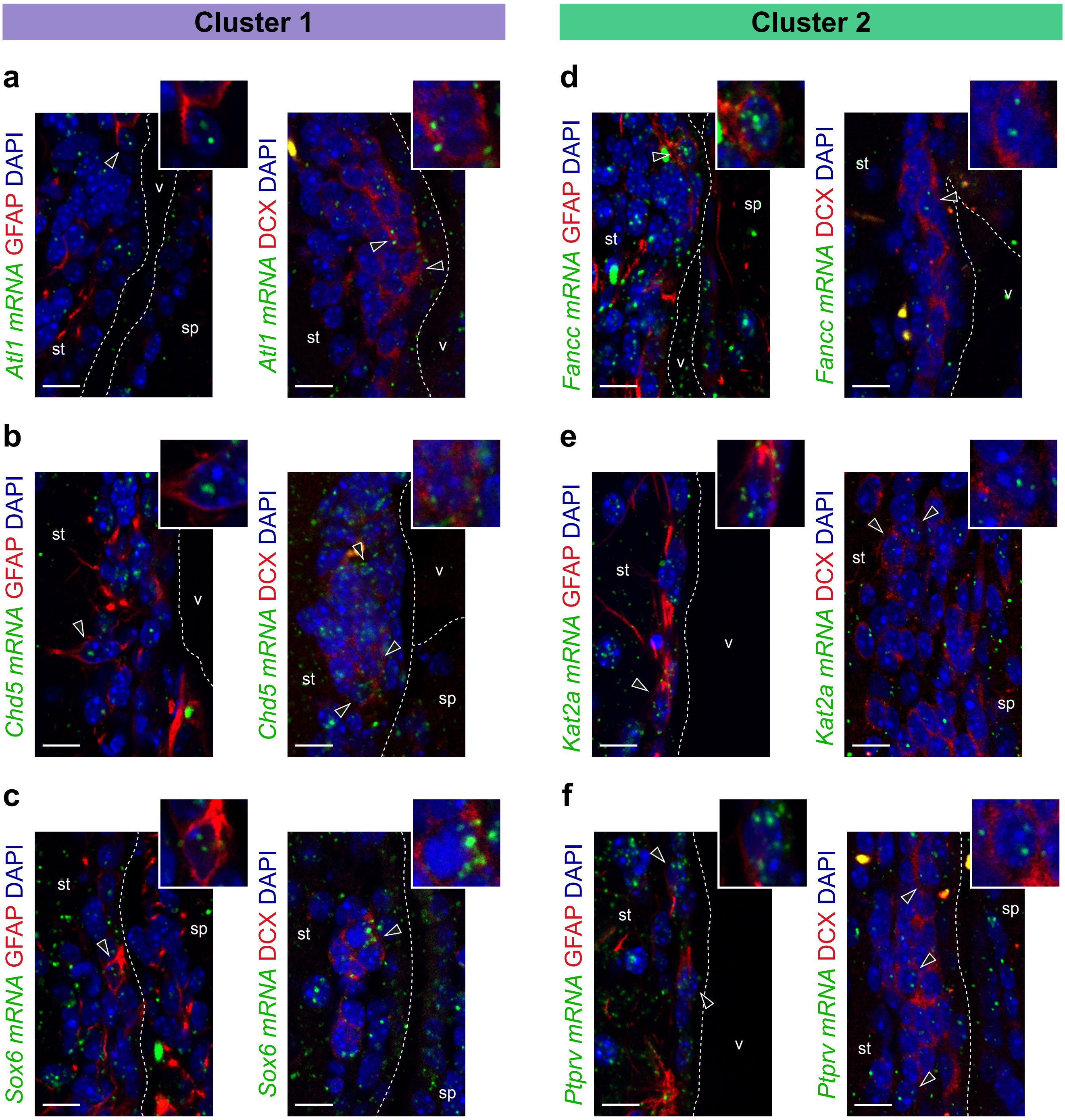

Supplementary figure 11| Distribution of Cluster 1 and Cluster 2 transcripts in NSCs and neuroblasts from the adult SEZ. a-c, *In situ* hybridisation for selected Cluster 1 mRNAs (green; *Atl1* (**a**), *Chd5* (**b**) *Sox6* (**c**)) in the SEZ of adult mice (coronal sections), combined with immunohistochemistry for GFAP (NSCs, red, left panel) or DCX (neuroblasts, red, right panel). **d-f**, *In situ* hybridisation for selected Cluster 2 mRNAs (green; *Fancc* (**d**), *Kat2a* (**e**) *Ptprv* (**f**)) in the SEZ of adult mice (coronal sections), combined with immunohistochemistry for GFAP (NSCs, red, left panel) or DCX (neuroblasts, red, right panel). Empty arrowheads point to double-positive cells. Scale bars represent 10 µm. sp, *septum*; st, *striatum*; v, lateral ventricle.

Supplementary figure 12

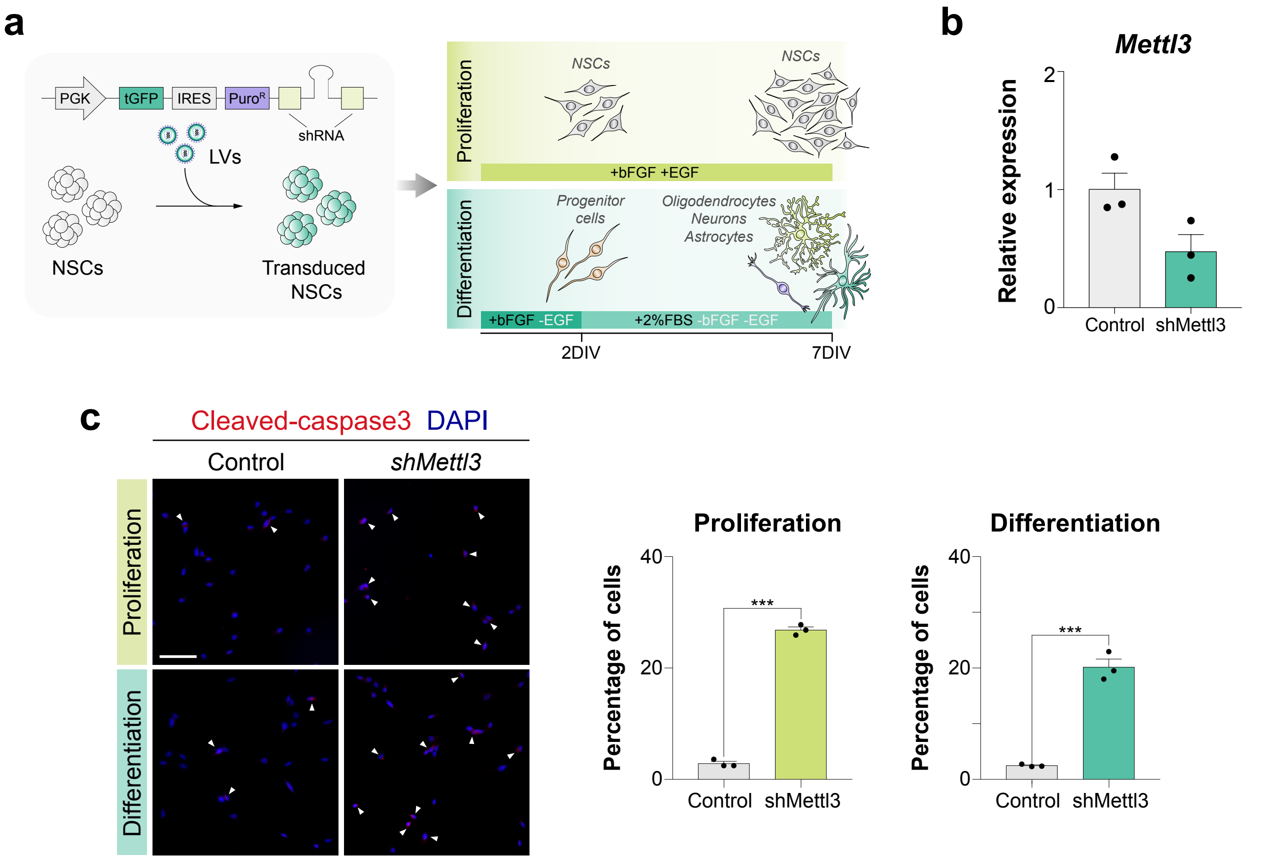

Supplementary figure 12| *Mettl3* loss of function causes an increase in apoptosis. a, Schematic representation of the transduction of NSCs with lentiviruses (LV) and the construct used for *Mettl3* loss of function experiments. Transduced NSCs were subsequently cultured either in proliferation or differentiation conditions. **b**, Analysis of *Mettl3* shRNA efficiency in NSC cultures by qPCR (n=3 biologically independent samples). **c**, Immunodetection and quantification of cleaved-caspase3^{+} cells (red) in cultures infected with control or shMettl3 lentiviruses, both under proliferation and differentiation conditions (*p*-value(proliferation)=0.000003, *p*-value(differentiation)=0.00276, n=3 biologically independent samples, by two-tailed Student's t-test). Arrowheads point to positive cells. Scale bars represent 25 µm. Data are presented as mean values ± SEM. ns, not significant; *p < 0.05, **p < 0.01, ***p < 0.001. Source data are provided as a Source Data file.

Supplementary Table 1

Antibody	Host	Dilution	Application	Provider	Cat. no.
Alexa Fluor® 488 anti-chicken	Goat	1:500	IF	Thermo Fisher	A11039
Alexa Fluor® 488 anti-rabbit	Goat	1:500	IF	Thermo Fisher	A11008
Alexa Fluor® 568 anti-chicken	Goat	1:500	IF	Thermo Fisher	A11041
Alexa Fluor® 568 anti-mouse	Goat	1:500	IF	Thermo Fisher	A11004
Alexa Fluor® 568 anti-rabbit	Goat	1:500	IF	Thermo Fisher	A11011
Alexa Fluor® 647 anti-rabbit	Goat	1:500	IF	Thermo Fisher	A27040
Alexa Fluor® 568 Streptoavidin-conjugated		1:500	FISH	Thermo Fisher	S11226
βIII-tubulin	Mouse	1:300	IF	Covance	PRB-435P
Cleaved-caspase3	Rabbit	1:1000	IF	Cell Signaling	9664S
Digoxigenin-AP	Sheep	1:1000	ISH	Roche	11093274910
Digoxigenin-POD	Sheep	1:500	FISH	Roche	11207733910
Doublecortin	Rabbit	1:1000	IF	Abcam	ab18723
GFAP	Chicken	1:600	IF	Millipore	ab5541
GFAP	Rabbit	1:300	IF	Dako	2033429-2
HuC/D	Mouse	1:1000	IF	Invitrogen	A21271
N^6-methyladenosine (m^6A)	Rabbit	1:50	RIP	Sigma	ABE572
Nestin	Mouse	1:4	IF	Hybridoma Bank	Rat-401
Nucleoporin	Mouse	1:500	IF	Abcam	ab24609
O4	Mouse	1:300	IF	Hybridoma bank	AB531796
p53	Mouse	1:1000	IF	Cell Signaling	2524
Vimentin	Rabbit	1:500	IF	Abcam	ab92547

Supplementary Table 1. List of antibodies used. IF, Immunofluorescence; ISH, *in situ* hybridisation; FISH, Fluorescent *in situ* Hybridisation; RIP, RNA immunoprecipitation.
