# Supplementary material for: Intron detention tightly regulates the stemness/differentiation switch in the adult neurogenic niche
Source: Nat Commun. 2024 Apr 2;15:2837. doi: 10.1038/s41467-024-47092-z (PMC10987655; doi:10.1038/s41467-024-47092-z)
Supplement: Supplementary file 3 — Description of Additional Supplementary Files [file 41467_2024_47092_MOESM3_ESM.docx]

Description of additional supplementary files

File name: Supplementary Data 1

Description: List of in situ hybridisation probes

File name: Supplementary Data 2

Description: List of qPCR primers used
