# Supplementary material for: Intron detention tightly regulates the stemness/differentiation switch in the adult neurogenic niche
Source: Nat Commun. 2024 Apr 2;15:2837. doi: 10.1038/s41467-024-47092-z (PMC10987655; doi:10.1038/s41467-024-47092-z)
Supplement: Supplementary file 6 — Reporting Summary [file 41467_2024_47092_MOESM6_ESM.pdf]

nature portfolio

Corresponding author(s): M. Angela Nieto

Last updated by author(s): Feb 12, 2024

Reporting Summary

Nature Portfolio wishes to improve the reproducibility of the work that we publish. This form provides structure for consistency and transparency in reporting. For further information on Nature Portfolio policies, see our Editorial Policies and the Editorial Policy Checklist.

Statistics

For all statistical analyses, confirm that the following items are present in the figure legend, table legend, main text, or Methods section.

n/a	Confirmed	
☐	☒	The exact sample size (*n*) for each experimental group/condition, given as a discrete number and unit of measurement
☐	☒	A statement on whether measurements were taken from distinct samples or whether the same sample was measured repeatedly
☐	☒	The statistical test(s) used AND whether they are one- or two-sided<br>*Only common tests should be described solely by name; describe more complex techniques in the Methods section.*
☒	☐	A description of all covariates tested
☒	☐	A description of any assumptions or corrections, such as tests of normality and adjustment for multiple comparisons
☐	☒	A full description of the statistical parameters including central tendency (e.g. means) or other basic estimates (e.g. regression coefficient) AND variation (e.g. standard deviation) or associated estimates of uncertainty (e.g. confidence intervals)
☐	☒	For null hypothesis testing, the test statistic (e.g. *F*, *t*, *r*) with confidence intervals, effect sizes, degrees of freedom and *P* value noted<br>*Give P values as exact values whenever suitable.*
☒	☐	For Bayesian analysis, information on the choice of priors and Markov chain Monte Carlo settings
☒	☐	For hierarchical and complex designs, identification of the appropriate level for tests and full reporting of outcomes
☒	☐	Estimates of effect sizes (e.g. Cohen's *d*, Pearson's *r*), indicating how they were calculated

Our web collection on statistics for biologists contains articles on many of the points above.

Software and code

Policy information about availability of computer code

Data collection	- Microphotographs were collected with Olympus FV10-ASW (v4.2c) for confocal images or Leica LasX software (v3.7.4.23463) for widefield images.<br>- Quantitative RT-PCR data were collected with Step One Plus software (v2.3).
Data analysis	- All image analyses were performed using NIH ImageJ Fiji (v1.49m).<br>- TargetM6A (http://csbio.njust.edu.cn/bioinf/TargetM6A) was used to perform m6A site prediction.<br>- RNA-seq: Sequencing quality was checked using FastQC (Babraham Institute). Read alignment and gene count were performed using STAR (v.2.5.3) against M. musculus genome assembly mm10 (GRCm38 build; Ensembl). Differential gene expression analysis was performed with DESeq2 (v.1.22.1) using a Wald Test and p-values were adjusted for multiple testing using Benjamini-Hochberg FDR correction.<br>- Functional enrichment analysis: We used the enrichR R package (v.2.1) to perform general gene functional enrichment analysis. Gene Set Enrichment Analysis (GSEA) in Reactome pathways was performed with ReactomePA R package (v.1.30.0), while gseGO and gseKEGG functions in clusterProfiler R package (v.3.10.0) were used to carry out GSEA of GO terms and KEGG pathways.<br>- Alternative splicing genome wide quantification was performed using VAST-TOOLS (v.2.0.2) against M. musculus mm10 (GRCm38 build; Ensembl) and with ASpli R/Bioconductor package (v.2.0.0). Cluster analysis was performed using hierarchical clustering with the heatmap.2 function in the R package gplots (v.3.0.1.1) (https://cran.r-project.org/web/packages/gplots/index.html), and soft clustering using the fuzzy c-means algorithm in Mfuzz R package (v.2.42.0).<br>- Mouse genome (GENCODE release M25, GRCm38) intron coordinates were extracted using the gtf2leafcutter.pl script from LeafCutter.<br>- Intron fasta sequences were obtained with getfasta from bedtools (v.2.27.1) and their GC content was calculated with nuc from bedtools (v.2.27.1). ggpubr R package (v.0.4.0) (https://CRAN.R-project.org/package=ggpubr) was used to generate intron plots and statistics (two-tailed t-test).

- Statistical analyses were performed using GraphPad Prism (v8.0.2).
- Images and data were processed for visualization with Adobe Photoshop (v19.0) and Adobe Illustrator (v16).

For manuscripts utilizing custom algorithms or software that are central to the research but not yet described in published literature, software must be made available to editors and reviewers. We strongly encourage code deposition in a community repository (e.g. GitHub). See the Nature Portfolio guidelines for submitting code & software for further information.

Data

Policy information about availability of data

All manuscripts must include a data availability statement. This statement should provide the following information, where applicable:

- Accession codes, unique identifiers, or web links for publicly available datasets
- A description of any restrictions on data availability
- For clinical datasets or third party data, please ensure that the statement adheres to our policy

- RNA-seq data have been deposited in the GEO database under the accession code GSE180806.
- As a reference we used the Mus musculus GRm38 (mm10) genome assembly and mouse genome annotation version M25 obtained from GENCODE.
- RNA-seq data from Baser et al., 2019 are available in the GEO database under the accession code GSE944991.

Research involving human participants, their data, or biological material

Policy information about studies with human participants or human data. See also policy information about sex, gender (identity/presentation), and sexual orientation and race, ethnicity and racism.

Reporting on sex and gender	No human research participantes were used in this study.
Reporting on race, ethnicity, or other socially relevant groupings	No human research participantes were used in this study.
Population characteristics	No human research participantes were used in this study.
Recruitment	No human research participantes were used in this study.
Ethics oversight	No human research participantes were used in this study.

Note that full information on the approval of the study protocol must also be provided in the manuscript.

Field-specific reporting

Please select the one below that is the best fit for your research. If you are not sure, read the appropriate sections before making your selection.

☒ Life sciences ☐ Behavioural & social sciences ☐ Ecological, evolutionary & environmental sciences

For a reference copy of the document with all sections, see nature.com/documents/nr-reporting-summary-flat.pdf

Life sciences study design

All studies must disclose on these points even when the disclosure is negative.

Sample size	No statistical method was used to predetermine sample size. Sample sizes were based on previously published experiments by us and others (see as examples: Domingo-Muelas et al., 2022; Porlan et al., 2014; Fazilaty et al., 2019; Naro et al., 2017) and our own un-published data.
Data exclusions	All experimental data were included in the manuscript. In those few cases where samples did not reach the expected quality standard (due to technical problems during processing or manipulation), they were discarded before data acquisition to avoid bias.
Replication	All experiments were independently performed at least twice, with the exception of the RNA-seq. In the latter case, three biological replicates were used, with 3 technical replicates each. All replication attempts were successful.
Randomization	In all cases experimental groups were obtained from the same NSC primary cultures, so there was no need for randomization.
Blinding	The experiments were performed objectively and did not require blinding.

Reporting for specific materials, systems and methods

We require information from authors about some types of materials, experimental systems and methods used in many studies. Here, indicate whether each material, system or method listed is relevant to your study. If you are not sure if a list item applies to your research, read the appropriate section before selecting a response.

Materials & experimental systems

n/a	Involved in the study	
☐	☒	Antibodies
☐	☒	Eukaryotic cell lines
☒	☐	Palaeontology and archaeology
☐	☒	Animals and other organisms
☒	☐	Clinical data
☒	☐	Dual use research of concern
☒	☐	Plants

Methods

n/a	Involved in the study	
☒	☐	ChIP-seq
☒	☐	Flow cytometry
☒	☐	MRI-based neuroimaging

Antibodies

Antibodies used

Primary antibodies:
- Mouse anti-βIII-tubulin (Covance, PRB-435P)
- Rabbit anti-Cleaved-Caspase3 (Cell Signalling, 9664S)
- Rabbit anti-Doublecortin (Abcam, ab18723)
- Chicken anti-GFAP (Millipore, ab5541)
- Rabbit anti-GFAP (Dako, 2033429-2)
- Mouse anti-HuC/D (Invitrogen, A21271)
- Mouse anti-Nestin (Hybridoma Bank, Rat-401)
- Mouse anti-Nucleoporin (Abcam, ab24609)
- Mouse anti-O4 (Hybridoma Bank, AB531796)
- Mouse anti-p53 (Cell Signaling, 2524)
- Rabbit anti-Vimentin (Abcam, ab92547)

Secondary antibodies:
- Alexa Fluor® 488 anti-chicken (Thermo Fisher, A11039)
- Alexa Fluor® 488 anti-rabbit (Thermo Fisher, A11008)
- Alexa Fluor® 568 anti-chicken (Thermo Fisher, A11041)
- Alexa Fluor® 568 anti-mouse (Thermo Fisher, A11004)
- Alexa Fluor® 568 anti-rabbit (Thermo Fisher, A11011)
- Alexa Fluor® 647 anti-rabbit (Thermo Fisher, A27040)
- Alexa Fluor® 568 Streptoavidin-conjugated (Thermo Fisher, S11226)

In situ hybridisation:
- Sheep Digoxigenin-AP (Roche, 11093274910)
- Sheep Digoxigenin-POD (Roche, 11207733910)

RIP: anti-N6-methyladenosine (Sigma, ABE572)

Validation

- βIII-tubulin: Belenguer et al., 2016; Chirivella et al., 2017.
- Cleaved-Caspase3: Scheffel et al., 2020; Yuan et al., 2020; Kesireddy et al., 2019; Marchetti et al., 2019; Fujino et al., 2019.
- Doublecortin: Bott et al., 2020; Wu et al., 2016; Wang et al., 2016.
- GFAP: Delgado et al., 2014; Porlan et al., 2014; Marques-Torrejon et al., 2013; Ferron et al., 2010.
- HuC/D: Wang et al., 2021; Kunst et al., 2019; Tse et al., 2018.
- Nestin: Belenguer et al., 2016; Chirivella et al., 2017.
- Nucleoporin: Kinosada et al., 2017.
- O4: Belenguer et al., 2016; Chirivella et al., 2017.
- p53: Bowling et al., 2018.
- Vimentin: Zhu et al., 2021; Simon et al., 2021; Peiris et al., 2014.
- Digoxigenin-AP: Acloque et al., 2008.
- Digoxigenin-AP: Acloque et al., 2008.
- N6-methyladenosine: Zheng et al, 2018.

Eukaryotic cell lines

Policy information about cell lines and Sex and Gender in Research

Cell line source(s)	Mouse embryonic stem cell line 46C (Sox1-GFP-IRES-pac knock-in) was purchased from PimCells (PCEMM01). HEK293T cell line was purchased from ATCC (ACS-4500).
Authentication	ES cell line was kept and used when it had few passages from the vendor original stock and did not require further authentication. HEK293T cell line was not authenticated.
Mycoplasma contamination	All cell lines were tested negative for Mycoplasma contamination.
Commonly misidentified lines (See ICLAC register)	No commonly misidentified cell lines were used in the study.

Animals and other research organisms

Policy information about studies involving animals; ARRIVE guidelines recommended for reporting animal research, and Sex and Gender in Research

Laboratory animals	Mus musculus:<br>All the experiments were performed using adult C57BL/6J wild-type mice between 2- and 4-months-old. Mice were bread and housed in a temperature-controlled room under 12h periods of light/darkness, and were reared on standard chow and water ad libitum.<br><br>Danio rerio:<br>All the experiments were performed using 6 months-old male zebrafish (AB strain, wild type), which were maintained at 28ºC under standard conditions.
Wild animals	This study did not involved wild animals.
Reporting on sex	Only male mice were used in this study.
Field-collected samples	This study did not involve samples collected from the field.
Ethics oversight	Housing and experimental procedures were conducted in strict compliance with the European Community Council Directive (89/609/EEC) and the Spanish legislation. Ethical protocols were approved by the CSIC Ethical Committee and the Animal Welfare Committee of the Institute of Neurosciences.

Note that full information on the approval of the study protocol must also be provided in the manuscript.

Plants

Seed stocks	*Report on the source of all seed stocks or other plant material used. If applicable, state the seed stock centre and catalogue number. If plant specimens were collected from the field, describe the collection location, date and sampling procedures.*
Novel plant genotypes	*Describe the methods by which all novel plant genotypes were produced. This includes those generated by transgenic approaches, gene editing, chemical/radiation-based mutagenesis and hybridization. For transgenic lines, describe the transformation method, the number of independent lines analyzed and the generation upon which experiments were performed. For gene-edited lines, describe the editor used, the endogenous sequence targeted for editing, the targeting guide RNA sequence (if applicable) and how the editor was applied.*
Authentication	*Describe any authentication procedures for each seed stock used or novel genotype generated. Describe any experiments used to assess the effect of a mutation and, where applicable, how potential secondary effects (e.g. second site T-DNA insertions, mosiacism, off-target gene editing) were examined.*
